# Mindset-Oriented Negotiation Training (MONT): Teaching More Than Skills and Knowledge

**DOI:** 10.3389/fpsyg.2018.00907

**Published:** 2018-06-05

**Authors:** Valentin Ade, Carolin Schuster, Fieke Harinck, Roman Trötschel

**Affiliations:** ^1^Institute of Psychology, Leuphana University of Lüneburg, Lüneburg, Germany; ^2^Institute of Psychology, Leiden University, Leiden, Netherlands

**Keywords:** negotiation, training, mindset, learning transfer, training effectiveness, sustainable integrative agreements

## Abstract

In this conceptual paper, we propose that both skill set development and mindset development would be desirable dimensions of negotiation training. The second dimension has received little attention thus far, but negotiation mindsets, i.e., the psychological orientations by which people approach negotiations, are likely to have a considerable influence on the outcome of negotiations. Referring to empirical and conceptual mindset studies from outside the negotiation field, we argue that developing mindsets can leverage the effectiveness of skills and knowledge, increase learning transfer, and lead to long-term behavioral changes. We introduce an integrative negotiation mindset that comprises three inclinations which complement each other: a collaborative, a curious, and a creative one. We also discuss activities that help people to develop and enhance this mindset both in and out of the classroom. Our general claim is that by moving beyond the activities of conventional negotiation training, which focuses on skills and knowledge, mindset-oriented negotiation training can increase training effectiveness and enable participants to more often reach what we define as sustainable integrative agreements.

## Introduction: Negotiation Training, Learning Transfer, and Mindsets

While negotiation training has become increasingly popular, critics claim that there is still room for systematic improvement in its effectiveness (Movius, 2008; Lewicki, 2014). At the same time, it is not quite clear at which level of effectiveness this kind of improvement would begin, as few studies have measured effectiveness in terms of long-term learning transfer from the classroom to the professional and private lives of course participants (Coleman and Lim, 2001). Rather, the focus of “[n]egotiation training evaluation tends to be short-term, aspectual, and piecemeal" (Coleman and Lim, 2001, p. 363). This target may strike one as inadequate because “[e]ven when people learn integrative negotiation skills, they have great difficulty transferring these skills to new tasks" (Moran et al., 2008, p. 100) and training “has to ‘stick’ over time in order to be effective" (Lewicki, 2002, p. 2).

Outside the negotiation field, researchers in different disciplines have examined the learning transfer from the classroom to the real world and shown that even skills that were acquired over an extended period of time are often not applied outside of class (Michalak, 1981; Baldwin and Ford, 1988; Cheng and Hampson, 2008). As Michie et al. (2013) argue, training interventions are often complex, and identifying which of the many interacting components of an intervention are effective is challenging. Based on similar arguments, Burke and Hutchins (2007), characterize “training transfer [as] a core issue for human resource development (HRD) researchers and practitioners focused on designing interventions that support individual, team, and organizational performance” (p. 263).

In this context, learning transfer is crucial because of the considerable costs involved. Beer et al. (2016) report that U.S.-based companies spent over $160 billion on employee learning in 2015 (In 2011, this number was, as Miller, 2012 notes, $10 billion lower). Researchers assume, however, that only 10 to 50 percent of this training resulted in behavioral changes (Burke and Hutchins, 2007). Beer et al. (2016) argue that when people learn something in a training, “[f]or the most part, the learning doesn’t lead to better organizational performance because people soon revert to their old ways of doing things.” For this reason, these authors compare the loss of resources due to ineffective training to “training robbery” (Beer et al., 2016, p. 51). Numbers and diagnoses such as these may be relevant for the negotiation field in particular. Laker and Powell (2011) report that at least anecdotal evidence suggested that the interpersonal abilities developed during soft-skills training, (which deal with interpersonal abilities such as negotiation,) were transferred to the job substantially less often than those developed in hard-skills training (which focus on technical abilities). In general, the scholars of skill training do not seem to agree on which best practices in their field could increase training effectiveness.

Although some negotiation researchers also emphasize the development of attitudes as a learning goal for training (e.g., Coleman and Lim, 2001; Zweibel et al., 2008; Cuhadar and Kampf, 2015), the implicit assumption often seems to be that the paramount learning goal is the development of a distinct skill set. Accordingly, the implicit underlying question that much of the basic and applied research of the negotiation field ultimately seems to address is how to best achieve this learning goal (e.g., Gist et al., 1991; Nadler et al., 2003; van Hasselt et al., 2008; Williams et al., 2008; Lang, 2009; Chapman et al., 2017). These studies have led to a deeper understanding of negotiation processes, contexts, and related issues. Thus, they have provided valuable insights for negotiation students and have contributed to the development of more effective negotiation training in important ways. However, it remains unclear whether the skill set approach holds untapped potential for substantial increases in negotiation training transfer.

In this paper, we approach training effectiveness by asking a different question. Instead of following the paradigm, we challenge the paramount role of the skill set by considering whether there might be other learning goals for negotiation training that deserve attention. Hence, we examine whether there are other dimensions in addition to skills and knowledge that training could or should target. Drawing on research from outside the negotiation field, we suggest that it is possible to increase the long-term learning transfer and thus the effectiveness of negotiation training by integrating mindset development into this training. Here, we focus on training in both professional and academic learning contexts.

Psychologists have examined and conceptualized mindsets since the early 20th century (Marbe, 1915). In recent years, Gollwitzer’s mindset theory of action phases (Gollwitzer, 1990, 2012) and Dweck’s growth mindset concept (Dweck, 2006) in particular have received manifold attention from academics and practitioners. Of those mindset studies that use interventions, most examine the immediate effects of mindset manipulations. For instance, Gollwitzer (1990, 2012) studies which mindsets people have in which phases of decision-making processes. Lately, however, an increasing number of authors claim that mindsets, due to their impact on the professional and private effectiveness of people, can – and possibly should – be trained (e.g., Aronson et al., 2002; Marshak and Grant, 2008; Kennedy et al., 2013; Paunesku et al., 2015; Okonofua et al., 2016). This suggests that mindsets can also be enduring psychological constructs shaped by learning experiences. By training certain well-chosen mindsets, they may become the default mindsets of individuals in various situations. Also, negotiation scholars have lately started to examine mindsets and have shown that participants in laboratory studies can be primed into mindsets that affect their cognitive, emotional, and motivational processes in the following situation (e.g., Harinck and De Dreu, 2008; Trötschel et al., 2011). We argue that effective mindsets can and should be trained also for real-life negotiation contexts. To the best of our knowledge, no prior research on how this can best be done has been published.

In this conceptual work, we use Rucker and Galinsky’s (2016) definition of a mindset as a “psychological orientation that affects the selection, encoding, and retrieval of information; as a result, mindsets drive evaluations, actions, and responses” (p. 161). As suggested by various authors (e.g., Bargh, 1994; Bargh and Chartrand, 2000; Gollwitzer, 2012; Rucker and Galinsky, 2016), processes related to mindsets automatically and unconsciously mediate and moderate pre-defined responses and behaviors as soon as the mindsets are cognitively activated in a specific social context. Thus, mindsets influence cognitive, motivational, and emotional processes and thereby affect the way individuals consciously and unconsciously approach and behave in specific social contexts. They make related knowledge, skills, attitudes, schemas, and associations salient.

With respect to negotiations, we assume that people approach the social context of negotiations with different cognitive mindsets (Trötschel et al., 2011). As shown by various negotiation studies, many people in Western societies hold specific cognitive beliefs, are characterized by behavioral tendencies, and display emotional responses when entering negotiations (Thompson and Hrebec, 1996). They may, for example, approach negotiations as if they were zero-sum games, overlooking even easily discernable opportunities to create value (fixed-pie bias; Thompson and Hastie, 1990; De Dreu et al., 2000) or assume that parties’ interests are diametrically opposed. Also, they may assume that people tend to behave competitively (Harinck et al., 2000), or they may fear or mistrust the other party (Butler, 1999; Kramer and Carnevale, 2001). This negotiation mindset, which can be referred to as a distributive negotiation mindset, may prevent successful outcomes of negotiations even if the negotiation parties have acquired and learned various integrative negotiation skills such as adding issues or trading concessions based on diverging preferences, interests, and expectations.

The mindset that people hold influences how they perceive negotiations, feel about their counterpart, and behave in social interactions. We believe that negotiation training needs to address participants’ knowledge [e.g., regarding the role of Best Alternative To a Negotiated Agreements (BATNAs), Fisher et al., 2012], their skills (e.g., to use logrolling), and their mindsets, i.e., their general psychological orientation toward negotiations. Appropriate mindsets, we argue, can leverage the benefits that people derive from their knowledge and skills. We claim that by including mindsets as a fundamental and integral part of the training, instructors can enable participants to change their psychological orientation toward negotiations at the cognitive, emotional, and motivational levels. In this way, participants are able to learn to effectively apply their acquired knowledge and learned skills in an automatic, unconscious, efficient, and effortless manner.

In addition to claiming that a mindset-orientation is likely to increase training effectiveness, we propose and conceptualize a specific negotiation mindset, the integrative mindset. Describing training activities that can be implemented both in and out of the classroom, we suppose that people completing a mindset-oriented negotiation training (MONT) are more likely to be effective in real-life negotiations and to apply their learning over a longer period of time than people who complete a training that focuses only on skills and knowledge. These suppositions are based, among others, on the aforementioned automation aspects of mindsets and the expected benefits of training activities that negotiators are encouraged to perform after having received their classroom training, which will be discussed further on.

## Moving From a Distributive to an Integrative Negotiation Mindset

Due to their education, socialization, and personal experiences, many people have already developed a specific psychological orientation toward negotiations (Thompson and Hrebec, 1996; Gelfand and Christakopoulou, 1999), which could be described as a distributive mindset. At the cognitive level, they tend to have the fixed-pie bias and are likely to focus on parties’ positions rather than interests (Fisher et al., 2012), claims rather than offers (Trötschel et al., 2015), and their own concerns rather than those of others (Carnevale and Pruitt, 1992). Negotiators with a distributive mindset also tend to polarize and over-simplify their perception of other parties, quickly considering everyone who is not a friend as a foe (Galinsky and Schweitzer, 2015). For instance, U.S. President George W. Bush declared, weeks after 9/11, that each country in the world now only had two options: “Either you are with us, or you are with the terrorists” (CNN.com, 2001). We assume that the distributive mindset also leads people to quickly identify how they are different from their counterparts, for instance in terms of interests, age, gender, education, ethnicity, wealth, home town, etc. At the emotional level, a distributive mindset is characterized by distrust in and envy of the other party and, if one is of lower social status and thus feels threatened, anxiety. At the behavioral level, a distributive mindset may cause people to be vigilant and consciously or unconsciously approach negotiations ready to either fight, flee, or freeze. During the negotiation process, the distributive mindset is characterized by competitive behaviors and strategies (as, e.g., described Harinck and De Dreu, 2004; Harinck and Ellemers, 2006), which lower the possibility of establishing integrative agreements. That distributive mindsets are quite common in Western cultures is, for example, suggested by many negotiation metaphors. In the English language, many of these metaphors are taken from the realms of (distributive) games, war, or fighting. As Young and Schlie (2011) put it, “[o]ur language is infused with talk of tactics, flanks, concessions, gaining ground, and winning” (p. 191).

We believe that a distributive mindset can be a limitation for negotiators. It can intensely stress negotiators and seriously harm the relationships they have with their counterparts. In addition, it may prevent them from using their knowledge and skills to the best of their ability and in the best interest of parties. In other words, the distributive mindset is likely to decrease the benefits that negotiators could gain from their knowledge and skills during integrative negotiations, as it will prevent them from applying what they have learnt.

We argue that practitioners could be more effective in their day-to-day practice if they were to approach negotiations with what we will subsequently refer to as the integrative negotiation mindset. People with this kind of mindset tend to be collaborative, curious, and creative, and these three tendencies are likely to complement each other. Drawing on the definition of the term mindset by Rucker and Galinsky (2016), we understand the integrative negotiation mindset as a psychological orientation that steers cognitive, emotional, and motivational processes in negotiations toward collaboration, curiosity, and creativity. This mindset, we suggest, can positively leverage the effectiveness of the negotiation skills and knowledge that people have and thereby help them maximize their long-term utility.

Negotiators with an integrative negotiation mindset are more likely than others to reach sustainable integrative agreements. These agreements have four characteristics. First, they create value, as they are built on opportunities to exchange issue-related concessions. These concessions can be based on diverging preferences (e.g., to logroll), interests (e.g., to identify compatible interests despite presumably opposing positions), and expectations (e.g., to use contingent contracts). Value can also be created by adding issues to the negotiation, which then also may be used for exchanging concessions. Second, based on Druckman and Wagner (2016) and Albin and Druckman (2017), we can assume that sustainable integrative agreements are more likely to be implemented, as they describe that implementation is moderated by how fair parties consider the outcomes and processes of negotiations. Third, sustainable integrative agreements involve low transaction costs (e.g., time, money, emotional energy; see Ury et al., 1988) when created and implemented. Fourth, sustainable integrative agreements are established in processes that tend to improve the relationship between parties. As, for instance, Lewicki (2002), Curhan et al. (2006), or Fisher et al. (2012) point out, many negotiations occur in the context of ongoing long-term relationships with partners in personal and professional lives, and, at the political level, with other nations. Often these relationships are much more valuable to the negotiators than the outcome of a specific negotiation.

We suggest that an integrative mindset is not only useful for obviously integrative negotiations, but also for apparently distributive ones. This is because the full integrative potential of a negotiation cannot often be directly and comprehensively identified. In these situations, being more collaborative, curious, and creative can, at times, allow negotiators to identify and exploit integrative potential that, at first, remains hidden. For example, in the peace negotiations between Israel and Egypt mentioned below, mediators helped both parties to eventually find compatible interests behind opposing positions after many years of conflict. Israel’s main interest was security and Egypt’s was sovereignty (Sebenius, 1992). Once these interests were identified, the parties were able to create value and finally reach an agreement by exchanging interest-based concessions. In addition, even if value has already been created (e.g., by logrolling based on diverging preferences of parties for the price and delivery date in a procurement contract for a car), this does not mean that there is no untapped integrative potential left (e.g., by logrolling based on the location of delivery and the length of warranty).

We also propose that an integrative mindset is not only beneficial during a negotiation, but can also help people identify integrative potential even if they are not yet negotiating. People with an integrative mindset tend to become aware of opportunities for deals such as in the following example: The gardens owned by Theresa and Tom are next to each other, and each of them has one fruit tree. Theresa has a cherry tree, which allows her to harvest in early summer, and Tom has an apple tree, which allows him to harvest in late summer. When both of them chat one afternoon, Theresa realizes that Tom and she do not only say that they both love cherries and apples, but also that they feel that they experience what economists, such as Kauder (2015), call a diminishing marginal utility of each type of fruit. While they greatly enjoy the first pounds of fresh fruits in a given season, the last pounds, eaten toward the end of the season, do not make them as happy as the first ones. Having adopted an integrative mindset, Theresa now initiates a negotiation by proposing that they share both the cherries and the apples. Tom happily agrees, as he, too, realizes that in this manner, they can both increase their individual utilities.

As suggested above, we propose that aim of MONT is to develop an integrative mindset that is characterized by three complementary inclinations: a collaborative, a curious, and a creative one. What all of the behaviors reflecting these inclinations have in common is that they are all oriented toward sustainable integrative agreements. However, they also differ insofar as each of them can be activated independently. In addition, each has the potential to be effective in its own right, depending on the issues, relationship, and phase of the negotiation. When the stakes are high, negotiators will ideally act based on all three inclinations. The related processes and behaviors may occur simultaneously, sequentially, or in another pattern, depending on the specific opportunities and challenges of a negotiation. In the following paragraphs, we discuss the relevance of each of these three inclinations and their meaning at the cognitive, emotional, and motivational levels. Here, we offer a tentative definition of the integrative mindset as a basis for more comprehensive theoretical and empirical studies on this construct in the future.

### Collaborative Inclination (Toward Creating Synergy)

We propose that at the cognitive level, the collaborative inclination involves consciously and unconsciously perceiving all negotiation parties as partners who pursue the goal of synergistically creating value while reaching a mutually satisfying solution. As Edgren and Barnard argue in their conceptual work on integrated care workers Edgren and Barnard (2012, 2015), collaborative people tend to acknowledge others as co-producers of value, rather than merely as receivers. They do so, for example, by thinking about the strengths and weaknesses of their counterparts. By considering how synergy with others can be created, collaborative people can seek to find a solution that is better than the one that each party could develop on their own (Covey, 1989). We suggest that a collaborative inclination can help negotiators to quickly identify common ground and aspects in counterparts that are similar to their own, such as a salient shared identity in terms of interests, values, age, gender, or education. In a field study on collocated and geographically distributed teams, Hinds and Mortensen (2005) found that shared identity moderated the effect that distribution had on interpersonal conflicts. At the emotional level, we hypothesize that the collaborative inclination involves good will, empathy, and a lack of fear regarding the other parties despite possibly strong disagreements on the subject. In addition, we suppose that if negotiators have a collaborative inclination, positive emotions such as satisfaction or joy often not only result from individual gains, but also from the value created collaboratively and the very fact that a good relationship has been established or fostered. That means that we assume negotiators with a collaborative inclination experience emotions that one may associate with friendship, rather than with conflict. Also, we suppose that synergy often happens when parties enjoy each other’s presence. At the motivational level, we assume that this inclination increases negotiators’ willingness to invest energy in joint work, show respect, listen carefully, provide other parties with information, and exchange offers rather than claims. We suppose that a collaborative inclination is beneficial for negotiators, as they, in most cases, depend on their counterparts in order to find sustainable integrative solutions.

We will first illustrate the collaborative inclination by using the example of two fictional characters: Michelle, who has completed a MONT, and Max, who has not. Michelle and Max are friends, and when they discuss where and how to spend a vacation together, a collaborative inclination can benefit Michelle for several reasons. First, because she has the ability to create and foster a positive relationship, Michelle is more likely to be trusted by Max. Hence, she can expect to retrieve more correct and comprehensive information. Second, because she regards Max as a co-creator of value, Michelle is more likely to consider Max’s original ideas, analytical skills, knowledge on possible destinations, and so on. Third, by working toward an agreement that clearly does not only benefit her but also Max, she increases the likelihood that he will appreciate the outcome and the process and fulfill his obligations. It is important for her that he does so; otherwise, she might have to travel alone or find a new partner for this trip.

### Curious Inclination (Toward Retrieving and Analyzing Information)

At the cognitive level, the curious inclination of people leads them to be more interested in retrieving and deeply processing relevant information. Relevant information concerns, for example, parties’ interests and priorities, the characteristics of the negotiated resources, and possible signs of cognitive biases on both sides. Examples of the characteristics of the negotiated resource are its divisibility, ownership, and expected value (Trötschel et al., 2014). Besides the fixed-pie bias discussed above, cognitive biases also include, for instance, confirmation bias (Nickerson, 1998) and anchoring bias (Tversky and Kahneman, 1975). Whereas confirmation bias leads people to selectively use information in ways that confirm their preconceptions, anchoring bias describes the tendency of people to let the first information they receive regarding an issue influence themselves to a disproportionate extent. Analyzing interests, priorities, resources, and signs of biases allows negotiators to gainfully relate characteristics of their current negotiation to general negotiation concepts and theory. This allows them to adjust their behavior in response to specific situations and in line with what they learnt in the past. At the emotional level, the experience of curiosity plays a role as an epistemic emotion that facilitates exploratory behavior (Litman, 2005; Trevors et al., 2017), especially when it comes to inconsistent, surprising pieces of information or complex problems. We hypothesize that negotiators with a curious inclination do not feel threatened by a sudden turn of events in negotiations but instead accept or even enjoy emotional responses such as surprise or amazement as integral part of the negotiation process. Therefore, we suppose negotiators with a curious inclination to be similar to people who generally enjoy traveling in that travelers often find pleasure in exploring new cultures. At the motivational level, we assume that negotiators who are eager to learn more about their counterparts are willing to pay close attention to verbal and non-verbal clues, even if it costs time and energy. As they truly want to understand their counterparties, we believe they will not only ask more questions, but also address the underlying motivations and crucial aspects of viable solutions. In order to retrieve information on interests (as opposed to positions), negotiators can, for example, ask why-questions rather than what-questions (Malhotra and Bazerman, 2008).

A curious inclination is beneficial in several ways. For instance, people who analyze the interests and positions of all parties might conclude that while parties may have opposing positions, their underlying interests are compatible with one another (as in the classical example by Follett, 1940, of two sisters’ interests in the peel and juice of an orange). In addition, negotiators who analyze resources can, at times, create value by dividing some resources into sub-resources that are of different value to the parties and then make the pie bigger by using logrolling (Trötschel et al., 2014). As Podziba (2014) puts it, when they are curious, a “steady stream of new understandings moves people beyond their long-held perspectives to foster productive negotiations and build innovative solutions” (p. 244). At the same time, being and staying curious and therefore listening carefully to other people can be difficult. As Covey (1989) argues, listening requires people to open themselves to others which also makes them vulnerable. Therefore, negotiating curiously requires confidence and often courage and may become easier as the sense of trust between the negotiation parties grows stronger over time.

In the case of Michelle and Max described above, Michelle’s collaborative inclination would encourage Max to be more willing to share (honest) information. Her curious inclination now helps her use this openness to retrieve valuable information. By asking questions, she is able to learn more about his interests (he loves Italy), resources (owns a brand new tent), creative ideas (proposes to try CouchSurfing for a couple of nights), and analyses (concludes from his Internet search that July is cheaper than August). Also, her curiosity leads her to analyze the information that she has collected. During this process, she realizes, for instance, that Max’s preference for location (Italy) seems to be stronger than his preference for accommodation (CouchSurfing). Based on this knowledge and again lead by her curious inclination, Michelle decides to challenge her assumption and asks Max if her impression of his preferences is correct.

### Creative Inclination (Toward Developing Multiple Options)

At the cognitive level, the creative inclination may allow negotiators to think “outside the box.” Sassenberg and Moskowitz (2005) argue that being “creative implies, by definition, the attempt to avoid the conventional routes of thinking and, therefore, the avoidance of the activation of typical associations” (p. 507). In other words, creative negotiators are open to information and potential solutions that are beyond the scope of previously identified issues. We hypothesize that at the emotional level, people with a creative inclination are more likely to immerse themselves in the situation and to trust their problem-solving abilities. This is because creativity seems to build on people being open toward change and on their trust that they will be able to cope with new environments or ideas. Hence, we assume that they are hardly afraid of proposing solutions that might be perceived as weak. In addition, these kinds of negotiators perceive being creative as fun and rewarding. They feel pride in coming up with new ideas and feel joy when given a chance to systematically create new perspectives. At the motivational level, we hypothesize that negotiators with a creative inclination are characterized by high intrinsic motivation with regard to the problem-solving process (as generally suggested in a study on creativity outside the negotiation field by Amabile, 1983), and they are more willing to invest more time and energy into generating unconventional solutions before choosing one. This is because we assume that creative people are more likely to enjoy developing ideas and hence would favor taking time for that purpose. A creative inclination, therefore, might be indicated by the high frequency and the long duration of playful searches for multiple integrative solutions.

The central role of creativity in negotiations has, for example, been highlighted by Kurtzberg (1998), Balachandra et al. (2005a,b), and Wheeler (2013a). Creativity is beneficial in negotiations because creating value often requires individualized solutions that are substantially more complex than splitting the differences or agreeing on other simple forms of compromise. The 1979 Egypt-Israel Peace Treaty, for instance, used interest-related concessions by establishing that the Sinai Peninsula would become a demilitarized zone of Egyptian territory. In this manner, Egypt was able to fulfill its main interest (sovereignty) and so was Israel (security; Sebenius, 1992). It has also been argued that in the ongoing Israel-Palestine conflict, the debate over a one-state or two-state solution might be reframed by the consideration of creative alternatives (Asseburg and Busse, 2016).

In the case of Michelle and Max, she would encourage him to join her in developing different options before they make a decision. While some of them might be rather conventional (e.g., rent an apartment in Nice), others could be original (e.g., visit three different locations, volunteer on an acquaintance’s organic farm, participate in a local 20 k run).

## How to Train a Mindset?

In addition to the goals of developing negotiation skills and knowledge, which are at the core of traditional negotiation training, we suggest supplementing training with the goals of integrative mindset development, integrative mindset transfer, and integrative mindset activation. Participating in a negotiation training is an important step in the development of an integrative mindset. However, it will, in most cases, not be sufficient for permanently establishing this mindset. The purpose of MONT interventions, then, is to begin the process of mindset development in training sessions in an effective manner and to provide participants with what they need to refine and transfer their mindset after the course and to activate and retain it during real-life negotiations when necessary.

To help participants achieve these goals, we propose several mindset-focused activities for negotiation training contexts. While participants perform many of these activities outside the classroom, especially those related to transfer and activation, they are at least partially initiated during the training sessions in the classroom. We also recommend providing follow-up supervision or coaching to facilitate learning transfer and to offer support when participants face challenges concerning activation. If this process is successful, participants are equipped with a more effective default mindset with which they approach negotiations. This mindset then automatically triggers curiosity, creativity, and collaboration if participants find themselves in the social context of a negotiation, which thereby allows them to apply the knowledge and skills that they have acquired in a negotiation training when appropriate.

### Activities for Mindset Development in the Classroom

During the official negotiation training sessions, which will often happen in a classroom, participants learn why the mindset that their course addresses is helpful. Instructors can provide them with examples and general information, such as empirical findings or theoretical approaches, as suggested in a study outside the negotiation field by Aronson et al. (2002). They might, for instance, use the example of a managing director of a political education NGO, whose curious inclination helps her to learn something about her counterparts’ preferences in a coordination meeting with public officials. Overcoming her initial fixed-pie assumption, she is able to identify logrolling potential. Another example deals with an investment manager of a wind energy investor with a creative inclination who finds a way to enlarge the pie by adding issues to a private equity deal. When discussing these or other examples, instructors can point out how a distributive mindset and related behaviors could prevent individuals from reaching these results. This technique of mentally contrasting the goal of implementing intended behaviors with realistic barriers has proven to be effective for those seeking to increase goal commitment and joint outcomes, especially in addition with concrete plans how to overcome these barriers (e.g., ‘if-then plans’; Kirk et al., 2011, 2013). Using this approach in the classroom, instructors can not only provide theoretical information on the integrative mindset, but also increase participants’ commitment to acquire and develop it. As some of methods proposed in the following, the technique of stressing the importance of the content is not limited to the content of mindsets. We propose that trainers emphasize that MONT is a holistic approach and that participants should acquire and develop a mindset rather only than specific tactics or assumptions related to this state of mind.

During classroom sessions, trainers can also contribute to this process by giving the participants opportunities to practice the new approach in negotiation role-plays (which often focus on cognitive training elements) and simple improvisation theater exercises (which include emotional, motivational, and cognitive training elements). Such repeated practice may help participants to develop their mindset if they and the instructors discuss this dimension before and after exercises. We recommend using classical negotiation exercises in which participants create and claim value, such as those published by the Kellogg School of Management’s Dispute Resolution Research Center (DRRC) or the Harvard Law School’s Program on Negotiation (PON). In order to train, for example, the use of integrative contingent contracts, the DRRC’s exercise Thai Solar Park (Ade, 2016) may be useful. While practice is an element central to skill-directed training as well, participants in MONT interventions are encouraged to activate an integrative mindset during the practice and, in this manner, behaviors are repeatedly linked to this mindset.

Less common in negotiation training, but promising for integrative mindset development, are improvisational exercises. As Harding (2004) and Balachandra et al. (2005a,b) argue, improvisation training can improve participants’ ability to intuitively recognize offers that they receive in negotiations, to be more explicit about what it is that they offer, and to use their counterparts’ offers as a base for formulating their offers. These authors also report that improvisation exercises improve several key skills associated with integrative mindsets, for instance, working together and co-creating results (collaboration), listening actively and picking up verbal and non-verbal cues (curiosity), and developing ideas (creativity). Balachandra et al. (2005a) conclude that “the incorporation of improvisation techniques into the negotiation skills repertoire holds great promise for practicing negotiators and is a worthy topic of future negotiation research and teaching” (p. 416). Koppett (2013), for example, provides a collection of improvisation exercises that may be valuable for negotiation training.

Classroom sessions likewise represent opportunities for participants to receive feedback from their peers and trainers. This feedback could, for instance, deal with the interests of counterparts during training negotiations. This kind of feedback has been shown to significantly improve negotiation abilities (Thompson and DeHarpport, 1994). In the classroom sessions of MONT interventions, feedback can also address not only others’ perceptions of a negotiator’s knowledge, skills, or behavior, but also the supposedly underlying mindset of the negotiator. We propose that by describing how they observe their peers, participants can help each other to develop a sense of the kind of mindset they may have. They can also learn, among other aspects, whether their mindsets tend to be stable during exercises and role-play.

During such role-play and exercises, it is beneficial if instructors prepare participants for real-life negotiations with parties that hold a distributive mindset. In these encounters, creating value will be more difficult, and participants who start the negotiation with an integrative mindset may experience frustration and might be tempted to switch to a distributive mindset. Anticipating such situations, practicing awareness of the other parties’ mindsets, and learning to accept failures when trying to maintain an integrative mindset can help participants to become more resilient in the face of perceived social pressure and powerful old habits such as switching to a distributive mindset.

It is important to point out here that having an integrative mindset can be fully compatible with behaviors that at first might appear to be non-collaborative, such as not trusting your counterpart or strategically withholding certain information. If a negotiator trusts an untrustworthy counterpart, this counterpart might sooner or later betray this negotiator. This may then lead to emotional and material harm and may cause the destruction of the relationship. So, effectively protecting oneself against betrayal can be necessary for protecting the relationship. Hence, having an integrative mindset might mean that negotiators, in some situations, may behave in what appears to be a resolute and confrontational rather than a soft and accommodating manner.

### Activities for Mindset Development and Transfer Outside the Classroom

In order to deepen their learning and improve learning transfer outside the classroom, participants can be instructed to reflect on their skill and mindset development by writing, reading, drawing, and talking with others. Writing reflective learning journals (as, e.g., recommended by Macduff, 2009 or Wheeler, 2015) is one way for participants to reflect on skill application, goals, motivation, and mindset-related experiences during the training and in real-life negotiations. As Balachandra et al. (2005b) argue, writing a journal can help negotiation students “become much more consciously aware of their *unconscious* strategies” (p. 437). Outside the negotiation field, reflective writing has been used in mindset interventions by Aronson et al. (2002); Paunesku et al. (2015), and others. It has also become an active research field in, to give but one example, medical education (Ng et al., 2015). Besides reflective journals, participants can increase their learning and motivation by reflecting on negotiation theory and practice while creating their own negotiation exercises (Ebner and Druckman, 2012; Druckman and Ebner, 2013; Wheeler, 2015). Doing so may foster participants’ identification with the learned skills and the related mindsets. If completed during the course, the exercises can be used and discussed in class.

Mindset transfer will be easier for participants if they receive external support. MONT instructors may want to point out why and how external support can be beneficial after the course and how it can be acquired. This kind of support may involve feedback from peers, a professional coach, or a ghost negotiator. These aides may provide negotiators with outside observations in a more comprehensive fashion than is often possible in classroom training. Ideally, these external supporters would focus on negotiators’ mindsets. For instance, an executive of a fashion retailer may hire a negotiation coach to prepare for a crucial negotiation dealing with a possible joint venture with the company’s main competitor. In the coaching meetings, the external perspective of the coach may help them to identify a tendency of the executive to be affected by the rivalry between the two companies and to switch to a distributive mindset. The executive and the coach can then reflect on this tendency together and role-play the negotiation, practicing maintaining the integrative mindset. Rehearsing a negotiation before it happens is, for instance, recommended by Ury (2007) and Diamond (2010). Diamond recommends that negotiators do not only to play their own role, but also gain a new perspective on a given situation by playing their counterpart.

Besides receiving external support in between and after training sessions, also providing such support for others may be beneficial for participants. Instructors may therefore want to point out that peer teaching what the participants have learned to others may help the participants in their own skill and mindset development (as suggested outside he negotiation field by Aronson et al., 2002). A form of pre-structured peer-coaching could, for instance, be a component of a MONT.

### Activities for Mindset Transfer and Activation in Real-Life Negotiations

Due to the complex and chaotic nature of negotiations, people may feel psychologically challenged by this social context (Wheeler, 2013a,b). As Lewicki (2014) argues, it is not clear whether today’s negotiation courses “prepare negotiators for the complex, ‘unscripted’ reality of negotiation experiences that usually diverge from the clear, simple, transactional, bounded role plays, and cases studied in class” (p. 500). Therefore, after having developed and strengthened their skills and mindset during various activities inside and outside the classroom, participants would be well advised to enhance their own – as well as their counterpart’s – mindset during a negotiation. To do so, they can use four techniques discussed in greater detail below: mindset energizers, role model visualization, and if-then plans. We recommend introducing all of these tools in the classroom so that participants will be familiar with them before they apply them to real-life negotiations.

#### Mindset Energizers

Mindset energizers are meant to activate a negotiation mindset that has, at least to some extent, already been developed. They are performed by issuing statements or asking questions. In the former case, participants tell their counterparts that they intend to live up to the mindset that they have adopted. In the case of the integrative mindset, they may, for example, state that they aim to lead the negotiation in a collaborative manner, that they are curious to learn more about their counterparts’ interests and preferences, or that they are eager to explore new ways and perspectives to create new solutions in the negotiation process. When using a mindset-energizing question, negotiators could ask whether their counterparts understand this negotiation as an opportunity to create value. They might also inquire about the other parties’ willingness to be collaborative, curious, or creative.

By asking such questions, negotiators may, we propose, not only foster their integrative mindset, but also encourage their counterpart to consider the possibility of adopting an integrative orientation. Negotiators have been shown to imitate and reciprocate their counterparties’ frames under certain conditions (De Dreu et al., 1994). As Brett et al. (1998) argue, the likelihood of contentious behaviors of counterparts, which are common for people with distributive mindsets, could be reduced when negotiators refrain from mirroring or reciprocating these behaviors and explicitly address their approach in this kind of situation.

#### Role Model Visualization

Negotiators can also strengthen a mindset during a negotiation by visualizing a (fictitious) role model that embodies the positive qualities of a given mindset. Discussing behavioral change, Bandura (1977) argues that observing others succeed who are similar to oneself can raise one’s self-efficacy. White et al. (2017), in a study on 4- and 6-year-old children, suggest that self-distancing can improve perseverance. The researchers found that children who impersonated fictitious exemplar others, such as Batman or Dora the Explorer, showed more perseverance during a tedious task than children who did not impersonate others. Studies have shown that human role models can also provide self-enhancement and be a source of inspiration (Lockwood and Kunda, 1997; Lockwood et al., 2002). As Marx and Roman (2002) argue, role models can buffer the performance of women in math tests from certain debilitating effects. Drawing on these insights, we claim that by visualizing a role model that embodies the qualities of a mindset, negotiators can increase the motivation to act accordingly. In addition, negotiators can intuitively get a feeling for how to act without having to resort to the theory they learned and the skills they acquired. At the same time, visualizing a role model will affect their emotions (e.g., by making them feel secure and strong). A role model for negotiators developing their integrative mindset could, for example, be a public figure such as a politician who may have won the Nobel Peace Prize or secured a mutually benefiting industry pact between a labor union and employer representatives. In a challenging negotiation, people may visualize one of these public figures and ask themselves, “How would she or he act now”? Negotiators may also develop fictitious role models and visualize these simplified embodiments of the mindset. In general, role model visualizations may be initiated by cues that negotiators bring to negotiations. They may, for instance, have a drawing or a photo of their role model on their negotiation writing pad. Also, they may wear a jacket, ring, or a watch that reminds them of their role model. In a set of empirical studies on priming outside the negotiation field, Dijksterhuis and Van Knippenberg (1998) suggest that priming stereotypes or traits can bring behavior in line with these stereotypes and traits. As mentioned above, negotiation studies using priming have shown that people can be primed into certain mindsets (Harinck and De Dreu, 2008; Trötschel et al., 2011). Role model visualization is particularly relevant to the mindset approach because similar to a personality, the mindset is a construct that predicts behavior across various types of negotiations. Therefore, a mindset role model can provide guidance especially in unknown situations.

#### If-Then Plans

In order to increase the probability that a certain mindset is applied during real-life negotiations, lecturers can also ask participants to develop if-then plans for behaviors associated with that mindset. If-then plans, which specify a critical situation in the if-part and intended behaviors in the then-part, have been shown to be very effective when people seek to achieve goals that require overcoming habits (Gollwitzer and Sheeran, 2006). Similar results have been reported concerning integrative negotiating behaviors (Trötschel and Gollwitzer, 2007; Kirk et al., 2013). The successful application of this approach in this context is likely due to the fact that plans can automatize the initiation of intended behaviors. In order to facilitate the adoption of an integrative mindset, one could, for example, initiate alternative behaviors in situations that often trigger a distributive mindset (e.g., IF I have to make a decision with someone else, THEN I will ask them what they want to achieve and why). One could also link dimensions of the integrative mindset to internal triggers (e.g., IF I get frustrated, THEN I will focus on finding creative options). In addition, it would be possible to cue the use of energizers or role model visualization (e.g., IF my counterpart insists on his or her position, THEN I will state my wish to find an integrative agreement; IF I see an angry face, THEN I remember my role model). If-then plans are a particularly effective technique for MONT interventions because mindsets are largely meant to affect behavior automatically in various situations that have negotiation characteristics. Therefore, the if-then plan does not need to specify the specific most effective behavior, but rather behaviors that activate the mindset whenever it is useful. A similar effect as by if-then plans may also be achieved by using goal primes (as, e.g., proposed by van Koningsbruggen et al., 2011).

## Discussion and Conclusion

In this paper we argue that mindset development could and should be one of the key goals of negotiation training. We introduce the concept of an integrative negotiation mindset and present a wide range of activities that could be used in training. This new approach to negotiation training is needed because several studies suggest that many of the teachings offered today are not as effective as initially assumed. Outside the negotiation field, mindset training has already shown promising results. In our own work as negotiation lecturers, trainers, and coaches, we have found anecdotal evidence suggesting that the teaching of skills and knowledge is not sufficient for ensuring that course participants use these skills when beneficial.

### Innovative Nature of MONT Approach

We believe that MONT represents an innovative approach, as most studies on negotiation training primarily deal with skills and knowledge, although some authors also address attitudes (e.g., Coleman and Lim, 2001; Zweibel et al., 2008; Cuhadar and Kampf, 2015). Teaching the integrative mindset that we propose here goes beyond teaching integrative strategies and tactics, as a mindset is more than a tool. Rather, the integrative mindset is a cognitive, emotional, and motivational orientation that automatically and often unconsciously guides negotiators toward collaboration, curiosity, and creativeness. Having adopted this orientation, negotiators are more likely to use, we argue, integrative strategies and tactics, and they do so more effectively and sustainably. In other words, they are more likely to achieve long-term results. As we suggest here, the integrative mindset functions as a lever for the skills and knowledge of negotiators.

For those who aim to integrate the MONT approach in their classrooms, we not only propose several activities, but we also recommend that negotiation training be extended beyond the in-class sessions in order to not only initiate mindset development, but to also foster mindset transfer and activation in real-life negotiations. After all, mindsets are like muscles: Their performance improves whenever you train them. Moreover, most people spend more time in real-life negotiations than in negotiation training classrooms. If they use part of these negotiations outside the classroom as an opportunity to learn and to further develop their integrative mindset, they can increase their negotiation effectiveness.

### Relation of Mindsets to Similar Constructs

The integrative mindset that we propose, and especially the aspect of collaborative inclination, shares some dimensions with what has been variously referred to as the cooperative mindset (Harinck and De Dreu, 2008), cooperative mental set (De Dreu and Nijstad, 2008), or cooperative motive (Deutsch, 2011). Still, our concept differs from these three in four respects. First, the integrative mindset proposed here not only contains an orientation toward a mutually beneficial outcome in a conflict, but also focuses on the co-creation of value (synergy). Second, besides focusing on finding an agreement, individuals with an integrative mindset are more likely to successfully implement an agreement, to keep transaction costs low, and to invest time and resources in relationships. Third, we propose that another benefit of the integrative mindset is not only that it psychologically prepares individuals for realizing integrative potential, but also that it may allow them to help counterparts to do the same. One way for individuals to foster an integrative mindset in a counterpart is by, asking mindset-energizing questions. Fourth, the MONT concept is based on a comprehensive definition of the term mindset, and it therefore differs from more specific concepts such as schema, belief, or attitude.

### Limitations and Future Research

We see three main limitations of the MONT concept that we would like to discuss briefly. First, we developed the concept (including the three inclinations) based on best practices discussed among negotiation instructors, our analyses of real life negotiations, the attempt to group reoccurring themes in the negotiation literature under umbrellas, and our own experiences as instructors and coaches. The concept therefore has yet to be tested empirically. To address this shortcoming of the present study, we are working on a project that shall present a scale for measuring people’s integrative mindset. Also, we are considering a laboratory experiment on integrative negotiations that in which we plan on using this scale to collect behavioral data. Apart from that, we encourage studies of the long-term success that MONT participants (and control groups, one of which receives a skill and knowledge-only negotiation training) have in terms of what we earlier referred to as sustainable integrative agreements. As discussed above, the definition of success here not only comprises performance in value creation, but also the likelihood of implementation, the level of transaction costs, and the impact that a negotiation has on the relationship between parties. To examine these issues in greater detail, not only laboratory experiments but also quantitative and qualitative longitudinal field studies are likely to be useful.

Second, the relationship between the three inclinations collaboration, curiosity, and creativity is complex and likely to change from context to context. Depending on the situation, each inclination may play a very different role in reaching sustainable integrative solutions, and it might be exceedingly difficult to determine the relative importance by objective means. Due to this, analyzing negotiations based on our proposal may lead different observers to different conclusions. Therefore, we hope to encourage alternative conceptualizations of the integrative negotiation mindset and also of other mindsets, which might be even more promising for negotiators. Hence, our proposed characterization of the integrative mindset is intended to be an initial reference for a debate of the benefits and characteristics of appropriate negotiation mindsets. Moreover, we hope that the mindset training activities that we present can be a fruitful starting point for practitioners and researchers who want to develop, test, and conceptualize MONT exercises.

Third, despite the fact that no other studies on mindset training for negotiation contexts currently exist, we believe that a number of lecturers, trainers, and coaches are already supporting their course participants and coaches in developing appropriate mindsets for negotiations. While our MONT approach is new to the literature, similar concepts may already exist in practice. If this is the case, our contribution to the field may not be entirely original. That said, we believe that if negotiation mindsets are already being trained, it is even more important to start providing a theoretical and empirical base for them and to begin a discussion on best practices and related issues. Hence, we encourage not only that these insights be shared but also propose empirical studies in which researchers reach out to lecturers, trainers, and coaches to collect, aggregate, and publish their insights into MONT interventions. It is not a goal of this paper to question the legitimacy of today’s negotiation training even though we cite sources that question its effectiveness. Rather, we want to raise the awareness for the role that an effective combination of knowledge, skills, and mindsets can play in negotiations.

In future empirical research on negotiation mindsets, it seems promising to study in which ways exact mindsets function as a moderator and mediator of skills and knowledge. That would mean exploring to which extent participants of trainings develop a specific mindset, which then triggers related behaviors (so an A→B→C effect) and to which extent mindsets influence the relationship between training and related behaviors (so stronger or weaker relationship between A and C depending on B). Also, it may be interesting to measure the extent to which the effect of the mindset itself is mediated and moderated by various factors, such as, the mindset of one’s counterpart.

A literature review that is more comprehensive than the one that we present might provide insights by looking for evidence that the distributive mindset, as we suggest, actually is the default setting for many individuals. Here, work by behavioral economists, and also studies that compare different cultures might provide valuable insights. Future research might also address the question in which ways negotiators shall or can become aware of which mindset they are having in a given moment. Besides thoughts that might typically be related to the integrative and the distributive mindsets, emotional or physical manifestations might also give negotiators hints about their current mindsets. Such indicators might be joy, anger, relief, excitement, unrest, frustration, hope, an increase of the heart rate, or feelings of defensiveness or bonding. In addition, it may be interesting to examine the extent to which constructs such as approach-avoidance motivation (e.g., discussed by Elliot and Thrash, 2002), promotion and prevention focuses (e.g., Higgins, 1998), or a process vs. an outcome orientation can add to our understanding of relevant negotiation mindsets. Finally, we also believe that examining the relation between negotiators’ curiosity and the actual depth by which they process information that concerns the integrative nature of negotiation contexts could provide relevant insights into how MONT interventions can best be structured.

## Author Contributions

All authors have substantially contributed to this study. VA has had the lead in developing our ideas and in writing our manuscript. CS has contributed to the development of our ideas. Also, she has proposed formulations for the manuscript and sources. FH has contributed to the development of our ideas and has proposed sources. RT has contributed significantly to the development of our ideas. Also, he has proposed formulations for the manuscript and sources.

## Conflict of Interest Statement

The authors declare that the research was conducted in the absence of any commercial or financial relationships that could be construed as a potential conflict of interest.
